# Medical studies at the University of Freiburg in retrospect – study conditions, study quality and skills acquisition from the perspective of graduates

**DOI:** 10.3205/zma001684

**Published:** 2024-06-17

**Authors:** Kevin Kunz, Hannah Köpper

**Affiliations:** 1 University of Freiburg, Faculty of Medicine, Office of the Dean of Studies, Academic Teaching Development, Freiburg, Germany

**Keywords:** graduate survey, study conditions, acquisition of skills, quality assurance, evaluation

## Abstract

**Background:**

As part of the MER*LIN* project (Medical Education Research – Lehrforschung im Netz BW), funded by the Federal Ministry of Education and Research, graduate surveys were carried out at the Medical Faculty of Freiburg from 2012-2020. This article will primarily address the question of how the study conditions and competence orientation in Freiburg are assessed and where there is still a need for optimization.

**Method:**

The surveys were conducted among graduates of human medicine at the Freiburg Medical Faculty 1.5 years after graduation. Participation was possible using paper and online questionnaires. The response rates were 36%-43%.

**Results:**

The study conditions were largely rated as good. There is a need for optimization, especially in the area of scientific work. The level of skills acquired was assessed as good to moderate. There were discrepancies between the level of competence achieved during the course of study and the level of competence required to start a career.

**Discussion:**

There is a need for development in terms of preparation for starting a career. Compared to the professionally required level of competence, self-assessment was worse in most competence domains. In Freiburg there are approaches to further promote the acquisition of skills during studies. In order to evaluate these developments and future changes in the context of studies, graduate surveys are relevant.

**Conclusion:**

Graduate surveys are suitable for generating data on the basis of which curriculum design can be carried out or which can be used to prepare for change processes. The surveys in Freiburg will therefore be continued and supplemented with new, needs-based questions.

## Zusammenfassung

**Hintergrund:**

Im Rahmen des vom Bundesministerium für Bildung und Forschung geförderten MER*LIN*-Projekts (Medical Education Research – Lehrforschung im Netz BW) wurden von 2012-2020 an der Medizinischen Fakultät Freiburg Absolvent*innenbefragungen durchgeführt. In diesem Artikel soll v.a. die Frage behandelt werden, wie die Studienbedingungen und die Kompetenzorientierung in Freiburg bewertet werden und wo noch Optimierungsbedarf besteht.

**Methodik:**

Die Befragungen wurden unter Absolvent*innen der Humanmedizin an der Medizinischen Fakultät Freiburg 1,5 Jahre nach Studienabschluss durchgeführt. Die Teilnahme war mit Papier- sowie Online-Fragebogen möglich. Die Rücklaufquoten betrugen 36%-43%.

**Ergebnisse:**

Die Studienbedingungen wurden größtenteils als gut bewertet. Optimierungsbedarf gibt es v.a. im Bereich des wissenschaftlichen Arbeitens. Das Niveau der erworbenen Kompetenzen wurde als gut bis mittelmäßig eingeschätzt. Zwischen dem im Studium erreichten und für den Berufseinstieg nötigen Kompetenzniveau zeigten sich Diskrepanzen.

**Diskussion:**

Entwicklungsbedarf besteht hinsichtlich der Vorbereitung auf den Berufseinstieg. Im Vergleich zum beruflich geforderten Kompetenzniveau fiel die Selbsteinschätzung in den meisten Kompetenzdomänen schlechter aus. In Freiburg gibt es Ansätze, um den Kompetenzerwerb im Studium weiter zu fördern. Um diese Entwicklungen und zukünftige Veränderungen im Kontext des Studiums zu evaluieren, sind Absolvent*innenbefragungen relevant.

**Schlussfolgerung:**

Absolvent*innenbefragungen sind geeignet, um Daten zu generieren, auf deren Basis eine Curriculumsgestaltung erfolgen kann bzw. die als Vorbereitung auf Veränderungsprozesse hinzugezogen werden können. Daher werden die Befragungen in Freiburg fortgeführt und um bedarfsgerechte neue Fragestellungen ergänzt.

## 1. Introduction

Graduate surveys are a suitable instrument for the profile and strategy development of universities [1], [2] and the data-based optimization of study programs and curricula [3]. They provide insight into student satisfaction, structure, framework conditions, quality of studies, skills acquisition and career entry [2], [4], [5], [6]. This is also reflected in the increasing importance both in terms of the frequency and regularity of the surveys conducted and in terms of their scientific or higher education policy value [5], [6], [7]. From 2012-2020, as part of the MERLIN (Medical Education Research – *Lehrforschung im Netz BW*) project funded by the Federal Ministry of Education and Research (BMBF), graduate surveys were conducted at the Medical Faculty of Freiburg, which made it possible to identify long-term developments (e.g. in skills development). The results of the surveys will also be used as a guide for the upcoming adjustments to the degree program to meet the requirements of the new *Medical Licensing Regulations (Ärztliche Approbationsordnung*, ÄApprO) and the new *National Competence-Based Catalogue of Learning Objectives for Undergraduate Medical Education (Nationaler Kompetenzbasierter Lernzielkatalog Medizin*, NKLM) [https://nklm.de/zend/menu], e.g. for the question of which competencies need to be better covered.

In this analysis, we refer to eight cohorts of graduates. The central question for this article is how the study conditions and competence orientation in Freiburg are perceived and in which areas there is still a need for optimization. A further focus is the comparison of study conditions and skills acquisition as well as the derivation of implications for future surveys.

## 2. Method

### 2.1. Implementation and statistical analysis

The Faculty of Medicine in Freiburg has been conducting surveys of graduates of human medicine and dentistry since 2009 to ensure quality and further develop the curriculum. In the period from 2012-2020, these were carried out as part of the BMBF-funded joint project MER*LIN* at the five medical faculties in Baden-Württemberg. Up to and including 2014 (class of 2012/13), there was a cooperation with *INCHER Kassel (International Center for Higher Education Research)* for this purpose. The 2014 graduating class was surveyed by the Rectorate as part of the University of Freiburg’s interdisciplinary graduate survey. The *Competence Center for Evaluation in Medicine* in Freiburg then took over the coordination, implementation and evaluation. The surveys were conducted annually. The graduates were surveyed approximately 1.5 years after graduation. Participation was voluntary and was possible with both paper and online questionnaires.

The graduates received an invitation by post with a link to the online survey, followed by reminder letters at intervals of around 4-6 weeks, each of which included the paper questionnaire as an additional option for participation. Up to 60% of participants took part using the paper questionnaire, 40% online.

The questionnaire used comprised seven thematic blocks with, among other things, information on *Abitur *(high-school) graduation grades and admission procedures *(before the degree program), the course of studies and doctorate, studies, study conditions and skills acquisition*, the *current occupational and employment situation, the relationship between studies and career* – with a self-assessment of existing skills in nine domains based on the NKLM 1.0 at two points in time – retrospectively at the time of graduation and at the time of the survey with the validated *Freiburg Questionnaire for the Assessment of Competencies in Medicine (Freiburger Fragebogen zur Erfassung von Kompetenzen in der Medizin,* FKM) [8] – socio-demographic data and free text comments on positive aspects and areas for improvement of the degree program.

The research was conducted in accordance with the Declaration of Helsinki and was approved by the Ethics Committee of the Medical Faculty of the University of Freiburg (446/16). All participants were informed in advance in writing about the course of the study and data protection and took part voluntarily.

The analysis was carried out using IBM SPSS Statistics (V.28), both descriptively and using inferential statistical variance analysis methods reporting corresponding effect sizes.

### 2.2. Sample

In the following, we refer exclusively to graduates of human medicine. In the survey period 2012-2020 with the graduating years 2010/11-2017/18, a total of 898 graduates from Freiburg took part in the survey, 68% of whom were female. The response rate was between 36%-43% (see table 1 (Tab. 1)).

## 3. Results

### 3.1. Study conditions

The study conditions were assessed on a five-point Likert scale from “1=very good” to “5=very poor”. The survey was conducted using individual items, some of which can be categorized thematically.

#### Evaluation of the study conditions

For the most part, respondents rated the study conditions as “good” or “very good” (see table 2 (Tab. 2), green). The conditions for scientific working methods in particular were assessed as “poor” or “very poor” (blue), teaching in the area of communication in the middle range (red).

The analysis of the evaluation over time using a single-factor analysis of variance (ANOVA) showed significant differences between individual years for the evaluation of the communication-related study conditions (p=.000-p=.012 with η^2^=.021 (“offers for the acquisition of communicative skills”) to η^2^=.042 (“passing on information/expert knowledge to members of other medical professions”)), the study offers for “oral presentation training” (p=.038, η^2^=.017), the study conditions in the area of teaching forms, methods and offers (p=.002-p=.014 with η^2^=.019 (“topicality of the methods taught”) to η^2^=.024 (“offers for the acquisition of learning strategies”, “use of modern teaching methods” and “breadth of courses offered”), “access to required courses” (p=.013, η^2^=.017) and “offers to acquire self-management skills” (p=.006, η^2^=.024).

#### Desired emphasis on study offers

In addition, the extent to which the addressed study offers should be part of the degree program was determined. Here, a very high to high desired emphasis was shown for all the offers addressed (see table 3 (Tab. 3)).

#### Comparison of assessment of and desire for study offers

**Figure 1:**
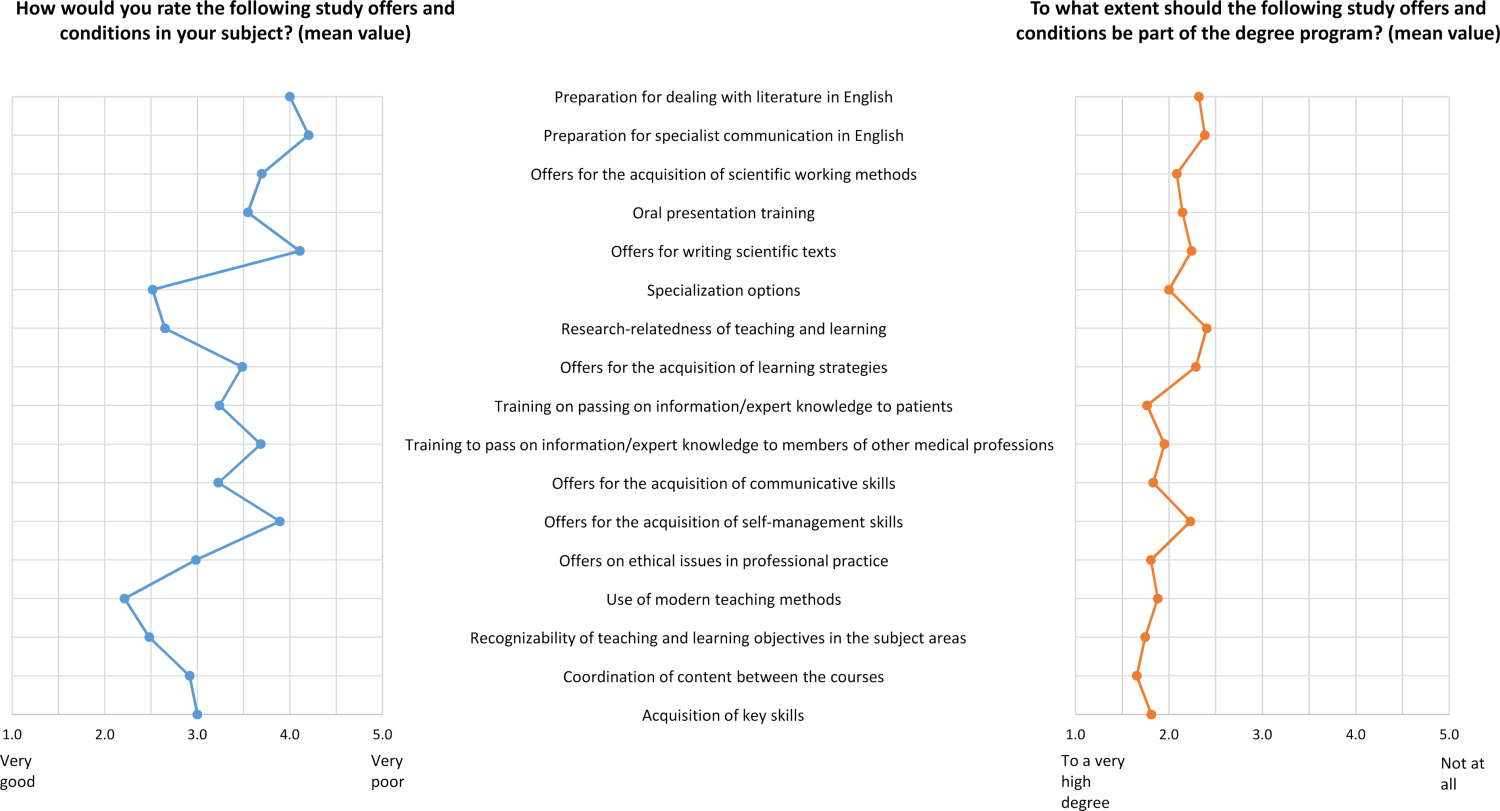
Assessment and desired emphasis of study programs and conditions

Figure 1 (Fig. 1) shows a graphical comparison of the assessment of study offers and conditions (blue) and the desire for which offers and conditions should be part of the degree program (orange). 

There were significant differences between the year groups with regard to the desired emphasis of the study offers. The calculated ANOVAs yielded significant results for “preparation for specialist communication in English” (p=.023, η^2^=.019) as well as for “preparation for dealing with literature in English” (p=.026, η^2^=.017). The desired emphasis continued to differ significantly between the year groups for “specialization options” (p=.005, η^2^=.024), the “research-relatedness of teaching and learning” (p=.018, η^2^=.019), “offers on ethical issues in professional practice” (p=.000, η^2^=.035), the “recognizability of teaching and learning objectives in the subject areas” (p=.046, η^2^=.017) and for “training to pass on information/expert knowledge to patients” (p=.031, η^2^=.018) and “to members of other medical professions” (p=.000, η^2^=.036).

### 3.2. Skills acquisition

The self-assessment of existing skills was carried out on a five-point Likert scale from “1=not at all” to “5=to a very high degree”. For the evaluation, individually surveyed items were combined into higher-level competence domains. In the upper range, the level of competence existing retrospectively at the time of graduation was assessed for the domains of *communicative competence* (aM=3.67, sd=.686), *learning competence* (aM=3.57, sd=.643), expertise (aM=3.50, sd=.475) and *competence in health promotion and prevention* (aM=3.35, sd=.670). Respondents rated the level of *team competence* (aM=2.96, sd=.790), *profession-related competence* (e.g. self-reflection, knowledge of ethical principles, involvement of patients in decision-making, ability to accept feedback) (aM=3.22, sd=.607) and *scientific competence* (aM=3.21, sd=.648) in the medium range.

**Figure 2:**
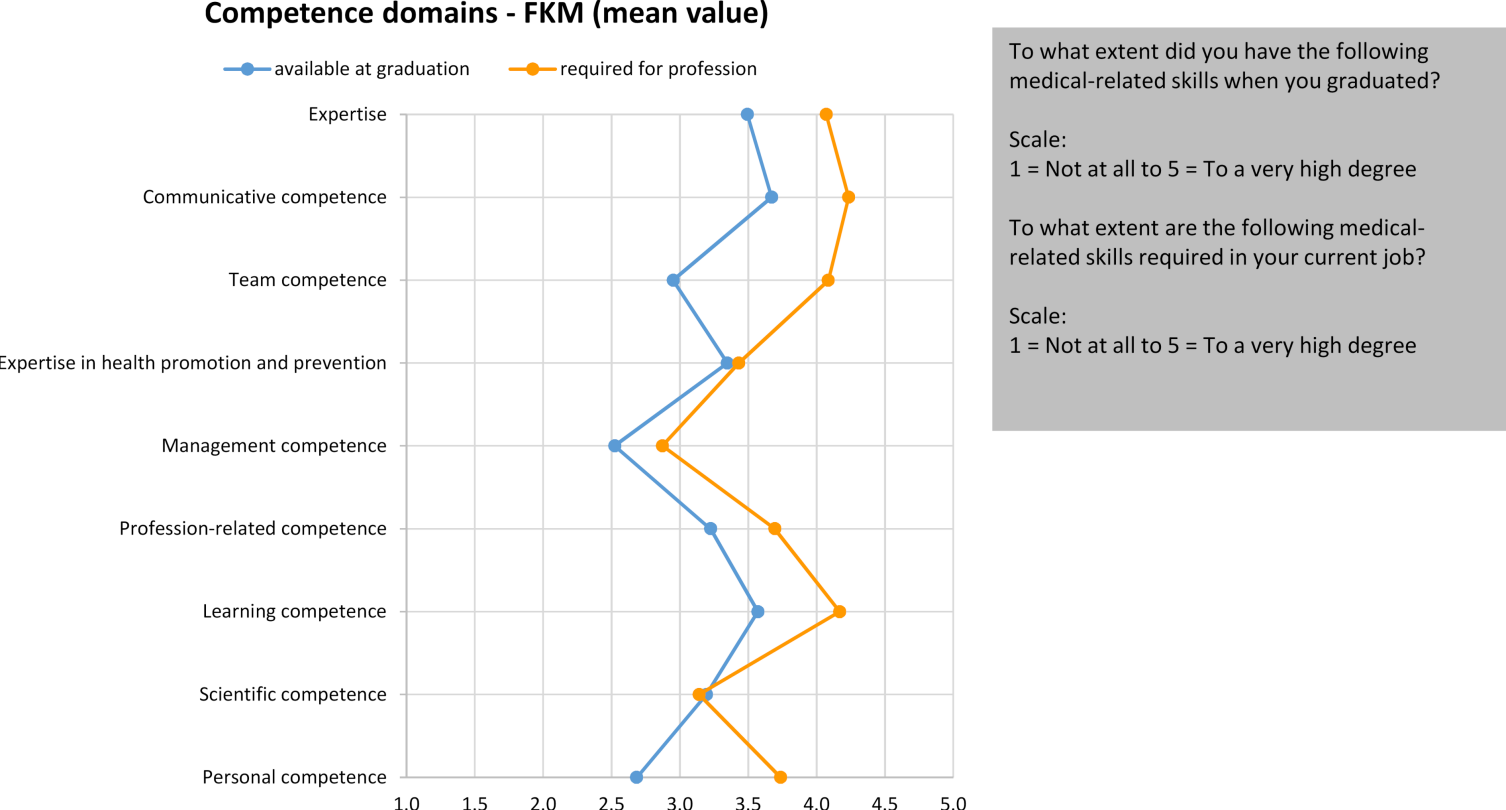
Competence self-assessment at the time of graduation compared to the competence level required in the profession

In addition, the FKM contains the assessment of the level of competence required in the profession [8]. The comparison of the information available in the data on the FKM using a t-test for paired samples showed that the respondents only considered themselves to be adequately prepared for entry into the profession in terms of *competence in health promotion and prevention* (p=.021) and *scientific competence* (p=.09). In the other domains, there were significant differences in relation to a higher requirement profile (p=.000, SRM=-1.09 to SRM=-.09). This difference was particularly clear in *team competence* (p=.000, SRM=-1.09) (see figure 2 (Fig. 2)).

The comparison of the individual year groups using ANOVA showed no significant differences with regard to the self-assessment of the level of competence in the domains surveyed, with the exception of *profession-related competence* (p=.008, η^2^=.038).

### 3.3. Student satisfaction

The majority of graduates were satisfied with their medical studies (15% very satisfied, 54% satisfied). 80% would choose the same degree program again. This high level of satisfaction persisted across all year groups.

## 4. Discussion

### 4.1. Discussion of the methodology

To conduct the survey, graduates were contacted approximately 1.5 years after graduation. This corresponds to a frequently chosen interval after graduation [5], [9]. The chosen time period offers the opportunity to ask questions about career entry and intended specialist training as well as retrospective evaluation of the degree program. The competence self-assessments are relevant in that the graduates have already gained their first professional experience 1.5 years after graduation and are therefore well able to assess the fit of the competences acquired during their studies with everyday professional practice [10].

The use of paper questionnaires had a positive effect on the implementation of the surveys - around 60% of total participation was via paper questionnaires. However, due to the high use of resources (financial, material, personnel) and the lower participation rate with paper questionnaires in the follow-up study (2020/21 cohort, 30% paper, 70% online), online-only participation is to be implemented in future.

The response rates of 36%-43% can be rated as good and correspond to the response rates of other graduate surveys [3], [10], [11]. Unfortunately, no statements can be made regarding the representativeness of the sample.

Despite all the advantages that graduate surveys offer, the informative value of the instrument should not be overestimated [4]. For example, the expertise of graduates must always be critically scrutinized with regard to the assessment of the skills acquired during their studies or required for the profession [12].

In our studies, we can largely compensate for the weaknesses mentioned by Teichler [13], but others unfortunately apply. For example, individual questions were changed in the course of the study (e.g. employment in rural areas). Overall, however, these adjustments remained minor and did not affect the questions analyzed for this report. A joint analysis and comparison of the surveyed graduation years is therefore possible and longer-term developments can be depicted well.

### 4.2. Discussion of the results

Overall, graduates rate the medical degree program and the study offers and conditions in Freiburg as good. Satisfaction with the degree program is high. Nevertheless, it became apparent that the respondents would like to see a stronger emphasis on almost all of the items related to the study offers and conditions. Particularly in the area of scientific training, it became clear that the respondents see the offers as an important part of the curriculum that should be strengthened, but only rated the study offers they experienced as poor to mediocre. A clear tendency in one direction of the evaluations, e.g. towards a clear improvement, is not recognizable. The effect sizes are in the small range. The significant results in the year group comparison could be due to strong differences in the group sizes compared. The same applies to the desired emphasis on the study offers.

The competence self-assessment showed that the respondents rated their competences at the time of graduation as good to average, i.e. there is still a need for optimization in the competence orientation of teaching. In comparison to the level of competence required in the profession, the self-assessment was worse in most domains. Depending on the competence domain, this discrepancy is likely to pose a challenge, particularly when entering the profession. Such survey data is relevant as it shows in which of the competence domains listed in the NKLM [https://nklm.de/zend/menu] there is still a need to catch up in terms of coverage during studies. Results from other graduate surveys also showed that respondents tended to rate their competencies as mediocre at the time of graduation, confirming the discrepancy found in other surveys between their own level of competence and the professional requirements [11].

Communicative competence was rated highest compared to the other competence domains surveyed. Across all year groups, a strong emphasis on study offers in the area of communication was desired. This was in line with the results from other surveys [14]. The importance of communication skills is also emphasized by the German Council of Science and Humanities [15], the Masterplan for Medical Studies 2020 (Masterplan Medizinstudium 2020) [16], the NKLM [https://nklm.de/zend/menu], other experts and students [17], [18]. In Freiburg, the topic is being promoted through a longitudinal communication curriculum and digital materials [19]. The effect of these measures on self-assessed skills acquisition is to be examined in future graduate surveys, among other things.

In Freiburg, as at other locations, the range of study offers for acquiring scientific skills was seen as capable of expansion [3], [11], [14], [20]. It was found that graduates would like to see a stronger emphasis on the study offers, but at the same time felt sufficiently prepared for their careers with the scientific skills they had acquired during their studies. However, this could also be due to the fact that young professionals in particular may not yet see scientific topics as relevant to the profession at this early stage and therefore consider their skills to be sufficient. In Freiburg, there are demonstrable improvements in the area of scientific skills. Since 2012, a curriculum for medical students has been developed and implemented as part of the work on the longitudinal curriculum of scientific skills with the aim of promoting scientific skills. So far, the level of competence in scientific skills has been assessed as mediocre. Other sources also report that the level of competence and teaching in the area of scientific skills is rated as poor and that there is a desire to strengthen the teaching of scientific skills [3], [17], [20], [21]. The German Council of Science and Humanities, the Masterplan 2020 and the Association of the Scientific Medical Societies in Germany (Arbeitsgemeinschaft der Wissenschaftlichen Medizinischen Fachgesellschaften, AWMF) also call for the handling of scientific skills to be practiced more during studies [15], [16], [22] and for “specific courses to strengthen scientific skills to be included in the compulsory curricula” [15]. In Freiburg, for example, a new lecture series has been introduced to strengthen the teaching of scientific skills.

The discrepancy between the assessments of the existing and required level of competence shows that there is still a need for action in the forthcoming revisions of the curriculum to cover some competencies in the degree program. Recent surveys conducted at the faculty among teaching staff and students confirm this. Such surveys should also be carried out in the future to supplement the graduate survey. The graduate surveys and other data can show, among other things, which NKLM competencies in the curriculum can still be developed or where the focus should be placed for optimization measures. This data is processed and used by working groups involved in curriculum development. As mentioned above, there are already initiatives in Freiburg in the areas of scientific skills and communication. In addition, the longitudinal curriculums of practical skills and interprofessionalism as well as a concept to promote digital skills are further initiatives being pursued to promote the acquisition of skills by students.

### 4.3. Implications for future surveys

The present results show that graduate surveys provide important information on the quality of studies, the coverage of teaching content and study offers, the acquisition of skills and preparation for professional practice. Against this background, the continuous implementation of graduate surveys is very important in order to be able to accompany future challenges, such as the new ÄApprO, the new NKLM, adjustments in student selection procedures or the focus on medical care in rural areas and other upcoming change processes [3].

The constantly changing challenges and circumstances require a continuous review of the suitability of individual questionnaire contents. For example, there is a need to revise the competency questionnaire in order to adapt it to the NKLM 2.0.

## 5. Outlook

The methodology of the graduate surveys has proven its worth. The data obtained should provide orientation for the challenges of the coming years, as we would like to show using the example of student selection, the *“rural doctor quota” (“Landarztquote”)* and skills orientation:

Changes in the legal conditions for student selection can have an influence on academic success. There is still little data available on this question. In view of the abolition of the waiting time quota and the greater importance of social skills in the selection process and graduate profile, these questions must be given more attention in future surveys. For example, the data may provide indications of the extent to which the academic success or later career path of people who gained access to studies through different selection procedures differs. The same applies to the so-called "rural doctor quota". By making adjustments to the questionnaire, it may be possible in future to make statements about which influences could lead graduates to see their future in a job in a rural region. In the medium term, it can also be shown what effects the adjustments to the curriculum as a result of the implementation of the new ÄApprO and the NKLM have on the perceived self-assessment of competence. Even before this, the data can provide an indication of the competence domains in which there is a need to catch up in teaching, so that these areas can be focused on in curriculum development. In order to answer these questions, among others, the questionnaire will be further optimized and supplemented with questions that also address other current political issues.

## 6. Conclusion

The results from the graduate surveys provide an insight into the assessment of study offers, study conditions, skills acquisition and career entry from the perspective of former students. By analyzing the data, important insights can be gained for the further development of the degree program. Graduate surveys should therefore continue to be used as an instrument for curriculum development and quality assurance.

## Funding

The graduate surveys were conducted as part of the BMBF-funded joint project MER*LIN* (Medical Education Research – *Lehrforschung im Netz BW*) of the Medical Faculties of Freiburg, Heidelberg, Mannheim, Ulm and Tübingen under the leadership of the Freiburg Medical Faculty. Funding reference: 01Pl12011A.

## Authors’ ORCIDs

- Kevin Kunz: [0009-0003-0563-6534]
- Hannah Köpper: [0000-0002-8995-7700]

## Acknowledgements

We would like to thank our cooperation partners in the MERLIN project for their constructive collaboration.

## Competing interests

The authors declare that they have no competing interests. 

## Figures and Tables

**Table 1:**
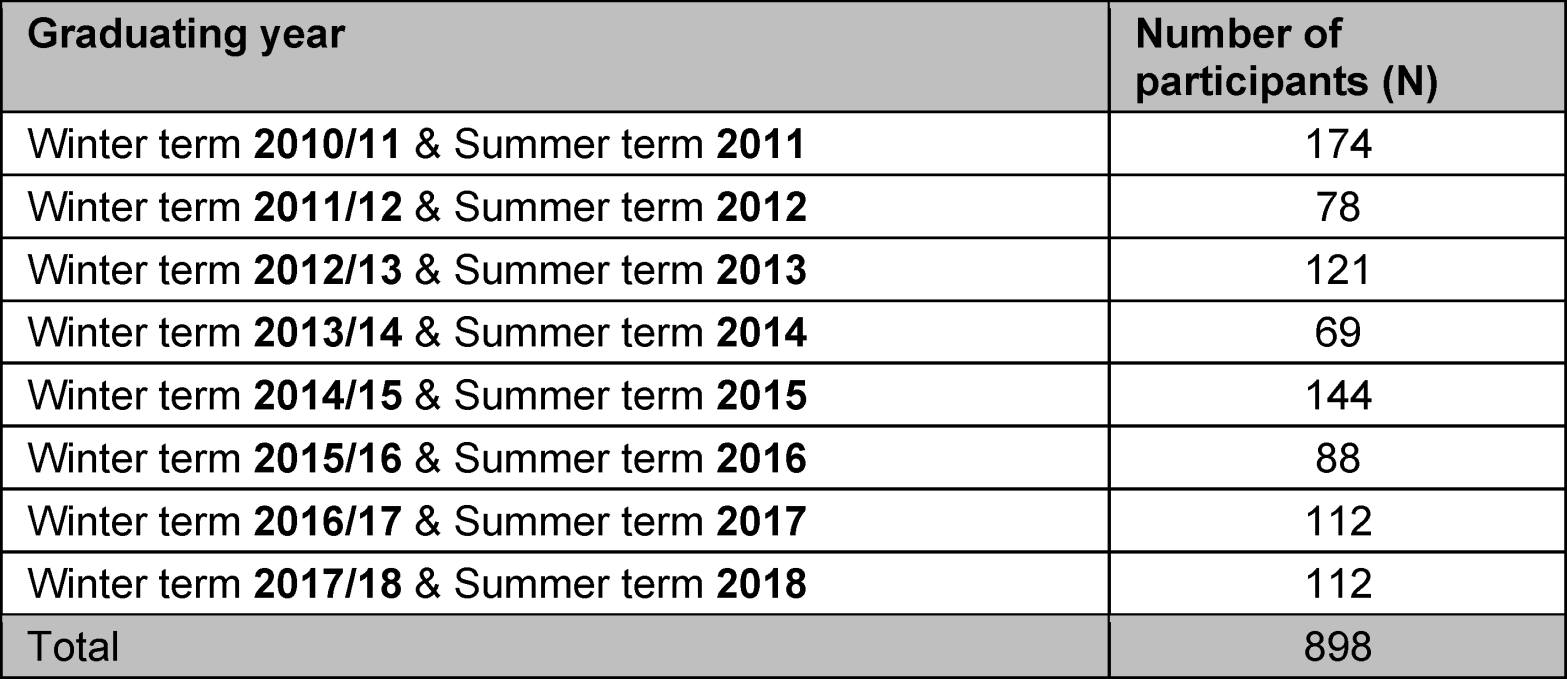
Number of survey participants per graduating year

**Table 2:**
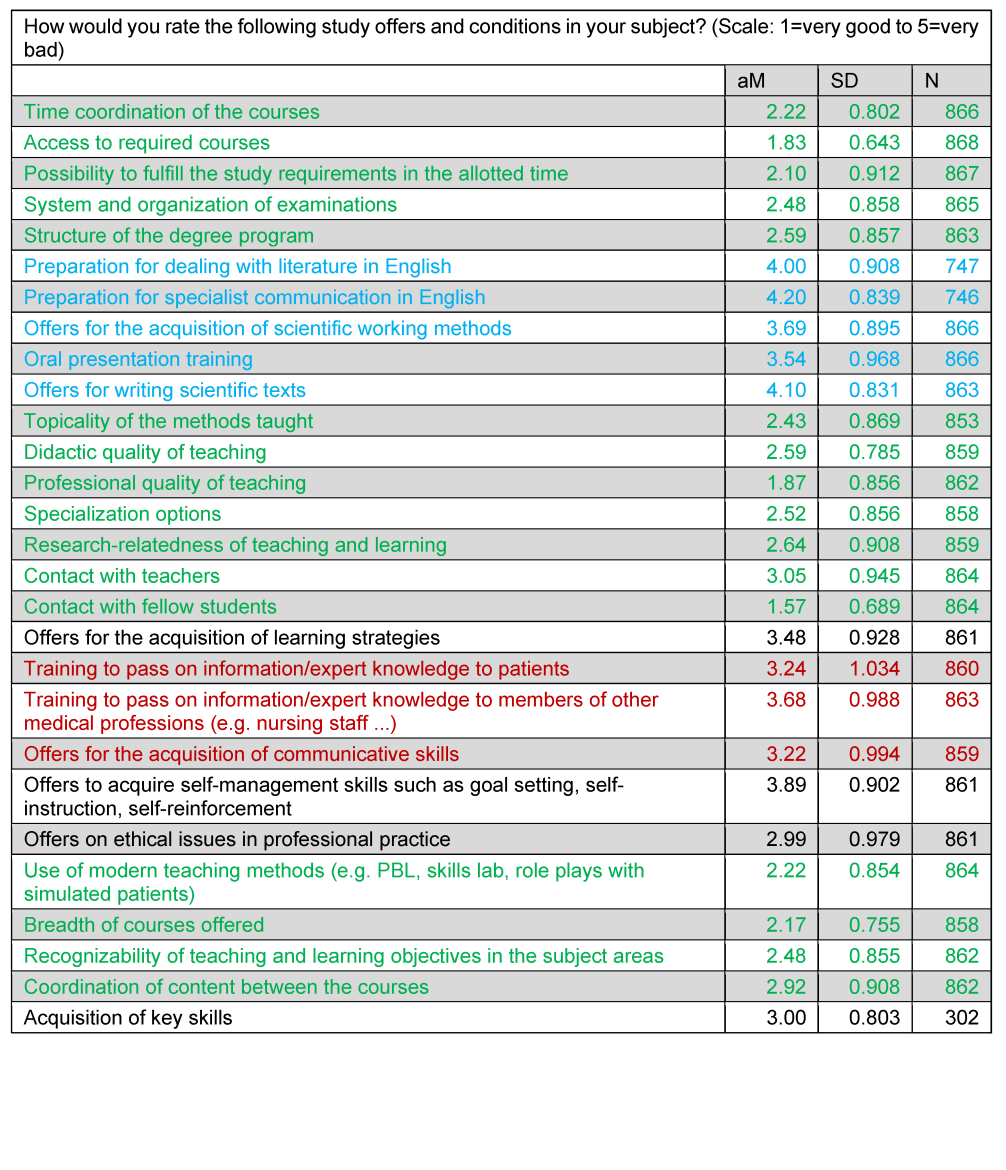
Assessment of study offers and conditions

**Table 3:**
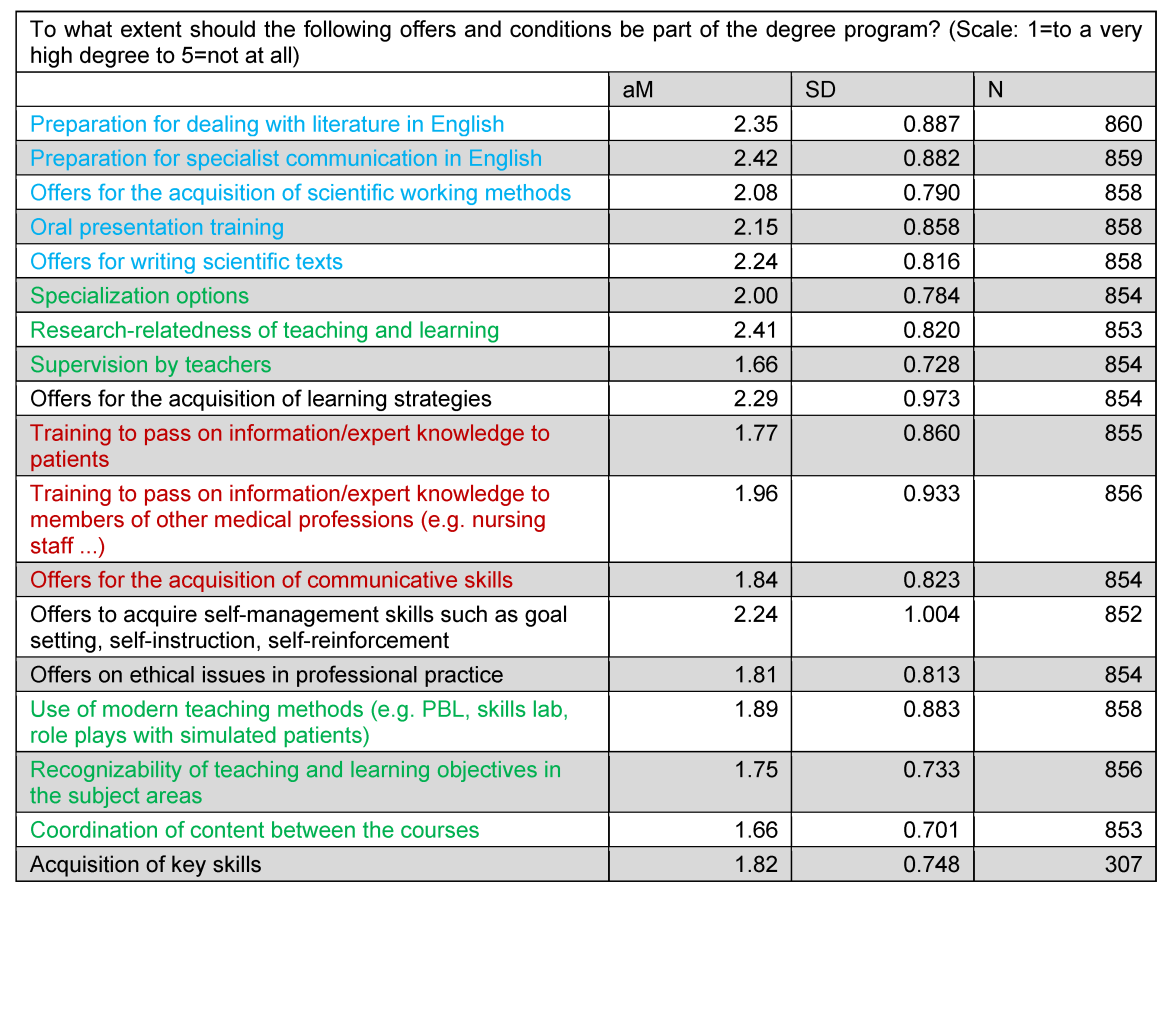
Desired emphasis on study offers and conditions
